# Involvement of *Plasmodium falciparum *protein kinase CK2 in the chromatin assembly pathway

**DOI:** 10.1186/1741-7007-10-5

**Published:** 2012-01-31

**Authors:** Eeshita G Dastidar, Guillem Dayer, Zoe M Holland, Dominique Dorin-Semblat, Aurélie Claes, Arnaud Chêne, Amit Sharma, Romain Hamelin, Marc Moniatte, Jose-Juan Lopez-Rubio, Artur Scherf, Christian Doerig

**Affiliations:** 1 Biology of Host-Parasite Interactions Unit, Institut Pasteur, 25 rue du Dr. Roux, F-75724 Paris, France; 2 CNRS URA2581, 25 rue du Dr. Roux, F-75724 Paris, France; 3 Inserm-EPFL Joint Laboratory, Global Health Institute, EPFL-SV-GHI, Station 19, CH-1015 Lausanne, Switzerland; 4 Wellcome Trust Centre for Molecular Parasitology, University of Glasgow, Glasgow G12 8TA, Scotland, UK; 5 International Centre for Genetic Engineering and Biotechnology, Aruna Asaf Ali Marg, 110 067 New Delhi, India; 6 Proteomics Core Facility, Ecole Polytechnique Fédérale de Lausanne, CH-1015 Lausanne, Switzerland; 7 Department of Microbiology, Building 76, Monash University, Wellington Road, Clayton, VIC 3800, Australia

## Abstract

**Background:**

Protein kinase CK2 is a pleiotropic serine/threonine protein kinase with hundreds of reported substrates, and plays an important role in a number of cellular processes. The cellular functions of *Plasmodium falciparum *CK2 (PfCK2) are unknown. The parasite's genome encodes one catalytic subunit, PfCK2α, which we have previously shown to be essential for completion of the asexual erythrocytic cycle, and two putative regulatory subunits, PfCK2β1 and PfCK2β2.

**Results:**

We now show that the genes encoding both regulatory PfCK2 subunits (PfCK2β1 and PfCK2β2) cannot be disrupted. Using immunofluorescence and electron microscopy, we examined the intra-erythrocytic stages of transgenic parasite lines expressing hemagglutinin (HA)-tagged catalytic and regulatory subunits (HA-CK2α, HA-PfCK2β1 or HA-PfCK2β2), and localized all three subunits to both cytoplasmic and nuclear compartments of the parasite. The same transgenic parasite lines were used to purify PfCK2β1- and PfCK2β2-containing complexes, which were analyzed by mass spectrometry. The recovered proteins were unevenly distributed between various pathways, with a large proportion of components of the chromatin assembly pathway being present in both PfCK2β1 and PfCK2β2 precipitates, implicating PfCK2 in chromatin dynamics. We also found that chromatin-related substrates such as nucleosome assembly proteins (Naps), histones, and two members of the Alba family are phosphorylated by PfCK2α *in vitro*.

**Conclusions:**

Our reverse-genetics data show that each of the two regulatory PfCK2 subunits is required for completion of the asexual erythrocytic cycle. Our interactome study points to an implication of PfCK2 in many cellular pathways, with chromatin dynamics being identified as a major process regulated by PfCK2. This study paves the way for a kinome-wide interactomics-based approach to elucidate protein kinase function in malaria parasites.

## Background

Malaria, a disease caused by parasitic protozoa of the genus *Plasmodium *(phylum Apicomplexa), is responsible for the death of one million people every year, mostly children living in sub-Saharan Africa [1]. The burden of morbidity and mortality inflicted by this disease presents a serious hindrance to socioeconomic development, causing up to a 1.3% decrease in the gross domestic product of countries with a high malaria rate [2]. The number of deaths due to malaria doubled in the last two decades of the 20th century, with one of the primary reasons being the spread of parasite resistance to anti-malarial drugs, especially chloroquine [3]. Artemisinin combination therapy (ACT) has helped to reverse this trend, but the worrying appearance in southeast Asia of parasite isolates with reduced artemisinin sensitivity suggests that this may be a short-lived respite [4,5]. The development of anti-malarial drugs with novel modes of action remains an urgent priority.

Reversible phosphorylation is an integral part of many cellular processes, and perturbations in its regulation have been identified in many diseases, such as cancer, diabetes and rheumatoid arthritis [6,7]. Protein kinases (PKs) are therefore attracting much interest as potential drug targets, and recent successes in cancer chemotherapy based on kinase inhibitors indicate that these molecules can perform as drugs with appropriate selectivity, potency and pharmacokinetic properties [8]. Investigations of the druggability of the genome identified serine/threonine PKs as one of the most promising groups for future small molecule inhibitors, comprising up to 20% of the druggable genome [9].

Protein kinase CK2 (thereafter called CK2) is a pleiotropic serine/threonine PK, with over 300 substrates identified in mammalian cells [10] and documented roles in multiple cellular processes, including differentiation, proliferation, survival, translation, apoptosis, transformation, tumorigenesis, RNA synthesis, cell cycle progression, cell morphology and polarity, cellular responses to stress and to DNA damage, and circadian rhythm [11,12]. The enzyme phosphorylates a range of DNA-binding proteins, nuclear oncoproteins, and transcription factors [13], and several studies have highlighted the predominance of CK2 substrates involved in transcription, chromatin structure, and gene expression [10,12,14]. Taken together, these studies highlight transcriptional regulation as one of the major roles of the enzyme [15]. Controlled cellular compartmentalization is crucial for the function of a pleiotropic enzyme such as CK2, and substrate selection is presumably dependent on proper targeting [12]. Although predominantly nuclear [16], mammalian CK2 has also been found in the cytoplasm, in association with a number of organelles such as the Golgi apparatus, endoplasmic reticulum, and ribosomes [17], and plant chloroplasts [18]. The human genome contains two genes encoding catalytic (α and α') subunits, and a single gene encoding a regulatory (β) subunit. CK2 is present in human cells as a tetrameric complex comprising two identical β subunits and two catalytic subunits, which can be assembled in a α/α, α/α' or α'/α' configuration. CK2α has been found to be essential for viability in all organisms in which its role has been assessed, including *P. falciparum *[19]. The requirement for viability of the β subunits of CK2 varies across species: disruption of the single metazoan β subunit is lethal in mice [20] and *Caenorhabditis elegans *[21], whereas disruption of either or both of the β subunits in *Saccharomyces cerevisiae *is not lethal [14,22,23].

Phylogenetic analysis of *P. falciparum *PKs [24,25] identified a CK2α ortholog and two putative CK2β subunits, PfCK2β1 and PfCK2β2 (see Holland *et al. *[19] for alignments with CK2 subunits form other organisms). The stoichiometry of the PfCK2 complexes is not known; by analogy with other systems, it is hypothesized that a homo- or heterodimer of β subunits is complexed with two catalytic subunits in the holoenzyme, but this still requires experimental demonstration. Using transcriptomics, it has been shown that all three subunits are expressed throughout the asexual erythrocytic asexual cycle, in gametocytes and in sporozoites [26,27]. Proteomics data available for PfCK2α and PfCK2β1 indicate that both proteins are present in the asexual erythrocytic stages and gametocytes, and that the α subunit is also present in sporozoites [28]. We showed previously that PfCK2α is essential for completion of the erythrocytic asexual cycle [19], but neither the requirement of the β subunits for viability nor the cellular processes implicating the enzyme have been assessed.

In the current study, we used a reverse-genetics approach, and found that both regulatory subunits are also essential for intra-erythrocytic asexual proliferation. Reasoning that identifying interactors of the regulatory subunits might provide useful insight into the cellular functions of the PfCK2 holoenzyme, we analyzed the protein composition of complexes obtained by co-immunoprecipitation of PfCK2β1 or PfCK2β2 from transgenic parasite lines expressing tagged subunits. This allowed us to recover proteins involved in many cellular processes, consistent with our immunofluorescence assay (IFA) data localizing the kinase subunits to both nuclear and cytoplasmic compartments. To investigate the role of CK2 in gene regulation specifically, we focused validation experiments on nuclear proteins, and found that several proteins involved in chromatin dynamics, which were recovered in the β subunit pull-downs, are substrates for PfCK2α *in vitro*. Taken together, our data identify PfCK2 as an essential kinase involved in chromatin dynamics.

## Results

### Both of the CK2β subunits play crucial roles in the erythrocytic asexual cycle

**Figure 1:**
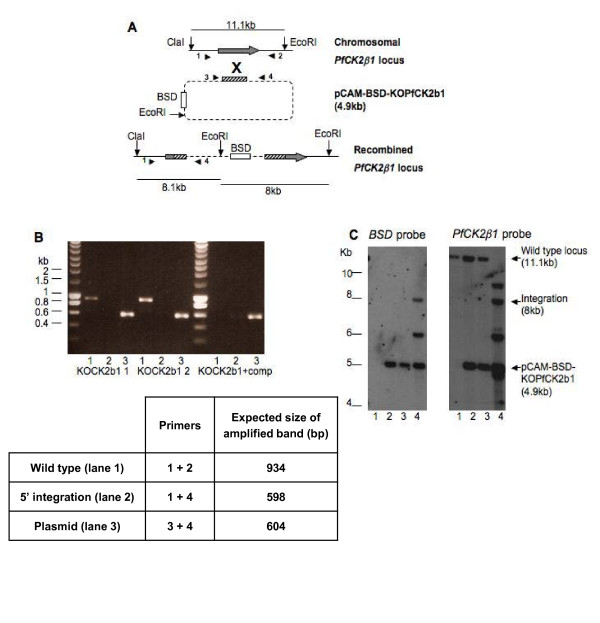
**Reverse genetics of PfCK2β1**. 3D7 parasites transfected with pCAM-BSD-KOPfCK2β1 with or without pCHD-PfCK2β1 were analyzed by PCR and Southern blotting. **(A) **Locations of the primers used for PCR screening, and the restriction enzymes used to cut the genomic DNA to give a diagnostic pattern of bands for analysis by Southern blotting. One truncated copy possesses its promoter and initiation codon, but lacks the C-terminal cysteine pair, a stop codon and a 3'UTR, whereas the other copy possesses both cysteine pairs but lacks a promoter and an initiation codon, and has an artificial stop codon introduced. **(B) **PCR screening of DNA from untransfected 3D7 parasites, two separate pCAM-BSD-KOPfCK2β1-transfected lines (KOCK2β1 1 and KOCK2β1 2), and parasites transfected with both the knockout plasmid and the complementation plasmid (KOCK2β1 + comp). (1) Amplification of the wild-type locus; (2) amplification over the 5' integration boundary; (3) amplification of the insert in the pCAM-BSD-KOPfCK2β1 plasmid. Evidence of integration was seen only in the DNA from the doubly transfected parasite culture (KOCK2β1 + comp; lane 2, faint band seen at 598 bp). **(C) **The parasite DNA was digested using the restriction enzymes *EcoR*I and *Cla*I, and analyzed by Southern blotting, using BSD and PfCK2β1 sequences as probes. (1) Untransfected 3D7; (2) KOCK2β1 1; (3) KOCK2β1 2; (4) KOCK2β1 + complement. See text for details.

We first aimed to determine whether PfCK2 β subunits play essential functions in parasite proliferation. CK2β proteins possess two conserved pairs of cysteine residues that form the base of a zinc finger required for homo- and heterodimerization [29], a prerequisite for CK2 holoenzyme formation [30]. To generate plasmids able to disrupt *pfck2β *genes, an internal fragment of the coding sequence excluding those crucial motifs was amplified and cloned into the transfection vector pCAM-BSD, which confers resistance to blasticidin [31]. It was expected that integration of the constructs (pCAM-BSD-KOPfCK2β1 and pCAM-BSD-KOPfCK2β2) into their cognate genomic loci by single crossover homologous recombination would result in a pseudo-diploid configuration, in which both truncated copies would be unable to express a functional protein (Figure 1A). After two independent transfections of pCAM-BSD-KOPfCK2β1 into 3D7 parasites, integration was monitored by PCR in the blasticidin-resistant populations (Figure 1B; see Additional file 1, table A1, for a list of all primers used in this study), using primer combinations that allow discrimination between the episome, the wild-type locus, and the disrupted locus. Only the episome (lane 3) and the wild-type locus (lane 1) were detectable, with no sign of integration even after prolonged culture (15 weeks). This might be due either to the fact that *pfck2β1 *is essential to the completion of the asexual parasite cycle, or to the possible refractoriness of the locus to recombination.

To ascertain whether *pfck*β*1 *can be disrupted if the subunit is provided through expression of an episomal copy of the gene, we constructed a complementation plasmid (pCHD-PfCK2β1) containing the full-length *pfck*β*1 *gene under the control of the *PfHsp86 *promoter and a 3' UTR. In parallel with the transfection of the pCAM-BSD-KOPfCK2β1 plasmid alone, a population of parasites was transfected with both pCAM-BSD-KOPfCK2β1 and pCHD-PfCK2β1. PCR (Figure 1B) and Southern blot (Figure 1C) analyses showed that disruption of the targeted locus occurred only in this doubly transfected, doubly resistant parasite culture. Probing the membrane with a *BSD *probe (Figure 1C, left panel) confirmed that integration had occurred only in the parasites transfected with both the knockout and complementation plasmids. The *pfck*β*1 *probe hybridized to the 11.1 kb band, corresponding to the wild-type locus, in the lane that contained DNA from untransfected parasites and from parasites transfected with the knockout plasmid alone (lanes 1 to 3). This band was undetectable in DNA from parasites transfected with both the knockout and complementation plasmid (lane 4), indicating that the gene can be disrupted only when an additional cassette coding for the PfCK2β1 subunit is provided to the parasites. There are multiple possibilities for recombination of the knockout and complementation plasmids before or after integration, which presumably accounts for the additional bands. The crucial observation is the disappearance of the wild-type-specific band only in the doubly transfected parasite culture. Similar results were obtained for PfCK2β2 (see Additional file 2, Figure A1), indicating that both regulatory subunits play crucial roles in parasite proliferation in the red blood cell.

### PfCK2 subunits localize to both cytoplasmic and nuclear compartments

**Figure 2:**
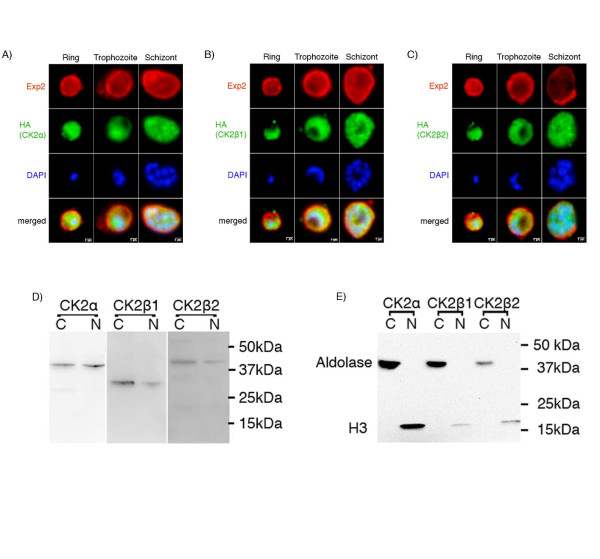
**Localization of PfCK2 subunits over the life cycle of the blood-stage parasite**. **(A-C**) Hemagglutinin (HA)-tagged PfCK2 subunits (green) localized to both nuclear and cytoplasmic compartments in rings, trophozoites and schizonts. The limits of the parasitophorous vacuole membrane (PVM) are marked by Exp-2 (red). **(D) **HA-tagged PfCK2 subunits were detected in both the cytoplasmic and nuclear fraction. Western-blot analysis was performed on the cytoplasmic (C) and nuclear (N) fraction of unsynchronized PfCK2α, PfCK2β1, and PfCK2β2 parasites using anti-HA antibody, which gave bands in both the fractions. Predicted molecular weights of the proteins are: PfCK2α, 39.8 kDa; PfCK2β1, 28.3 kDa; PfCK2β2, 45.2 kDa. **(E) **The same C and N fractions were probed with antibodies against aldolase, a cytoplasmic marker, and histone H3, a nuclear marker, to assess the purity of the fractions. Aldolase gave a band at 40 kDa in the cytoplasmic fractions only, whereas the histone H3 band at 17 kDa was seen only in the nuclear fractions.

**Figure 3:**
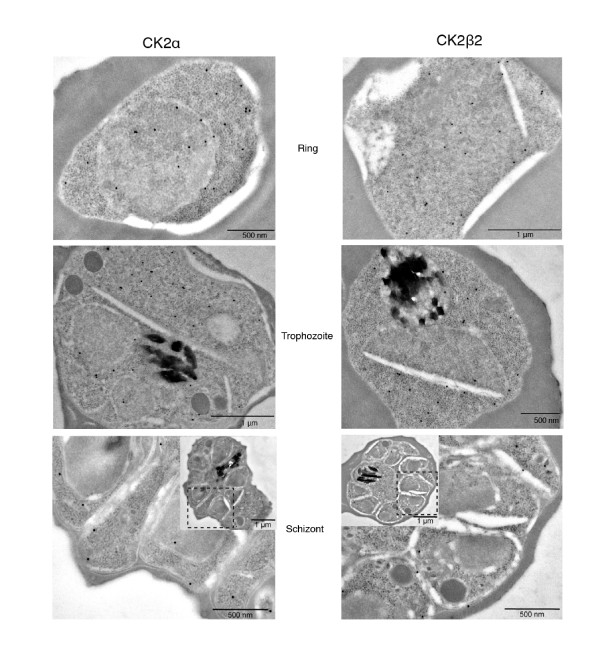
**Immuno-electron microscopy of the hemagglutinin (HA)-tagged PfCK2α and PfCK2β2 subunits**. Immuno-electron microscopy was performed with an anti-HA antibody on ring-, trophozoite- and schizont-stage parasites from the HA-tagged lines. HA-PfCK2α and HA-PfCK2β2 localized to both cytoplasmic and nuclear compartments in accordance with our IFA and western-blot data.

To generate parasite lines expressing tagged PfCK2β1 or PfCK2β2 from the endogenous loci, we transfected wild-type parasites with 3'-HA-tagged constructs (pCAM-BSD-HA-pfck2β1 or pCAM-BSD-HA-pfck2β2), which were expected not to cause loss of function of the target protein upon integration into the cognate locus. Integration of the tagging construct and disappearance of the wild-type locus were readily achieved for both genes (see 2 file 5, Figure A2, Figure A3). To determine the subcellular localization of HA-tagged PfCK2α [19], PfCK2β1 or PfCK2β2 subunits, we examined these transgenic lines in IFAs using antibodies against the HA epitope and against Exp2, a parasitophorous vacuole marker [32] (data for localization of all three subunits are presented in Figure 2A-C). In line with the pleiotropic nature of CK2 in eukaryotic cells, a signal for all three subunits was detectable in both the cytoplasmic and nuclear compartments of the parasite. The location of HA-tagged PfCK2α and PfCK2β2 in rings, trophozoites, and schizonts was further examined by immuno-electron microscopy, which confirmed presence of the protein in both cytoplasm and nucleus (Figure 3). Furthermore, bands of the expected sizes of all three subunits were clearly visible on anti-HA western blots in both cytoplasmic and nuclear fractions of unsynchronized cultures of the three HA-tagged parasite lines (Figure 2D). Antibodies against aldolase, a cytoplasmic marker, and against histone H3, a nuclear marker, were used to probe the same western-blot membrane to ascertain the purity of the subcellular fractions (Figure 2E). In conclusion, our data suggest that PfCK2 subunits are expressed throughout erythrocytic schizogony, and are present in various subcellular locations.

### Purification and mass spectrometry analysis of protein complexes involving the PfCK2β subunits

To gain insight into the functions of PfCK2 through identification of the proteins with which the holoenzyme interacts, we subjected protein extracts from the parasite line expressing HA-tagged PfCK2β1 to immunoprecipitation using the anti-HA antibody; the same analysis was performed on extracts from wild-type parasites as a negative control. The immunoprecipitates were separated by SDS-PAGE, and a western blotting was performed using anti-PfCK2β1 to verify the immunoprecipitation efficiency (see Additional file 2, Figure A4A). A parallel gel was loaded and slices excised (see Additional file 2, Figure A4B) for mass spectrometry analysis.

Potential interactors (see Additional file 3, Table A2) were identified based on the number of unique peptides recovered, and on the unweighted spectrum count of the number of detected peptides. We considered proteins as potential hits if they were present in the immunoprecipitate from the HA-tagged line but were absent (or present in trace amounts only) in those from the wild-type parasites. The same experiment was performed in parallel with the parasite line expressing HA-tagged PfCK2β2 (see Additional file 4, Table A3, for the list of recovered proteins), although in this case we were unable to perform western-blot verification of the immunoprecipitate because of the poor quality of the anti- PfCK2β2 antibody.

PfCK2α was pulled down with both HA-PfCK2β1 and HA-PfCK2β2, in line with the previously reported *in vitro *interaction between the catalytic subunit and both regulatory subunits [19]. Interestingly, PfCK2β1 and PfCK2β2 were present in similar amounts in both immunoprecipitates, suggesting that complexes containing both regulatory subunits may be relatively abundant.

**Figure 4:**
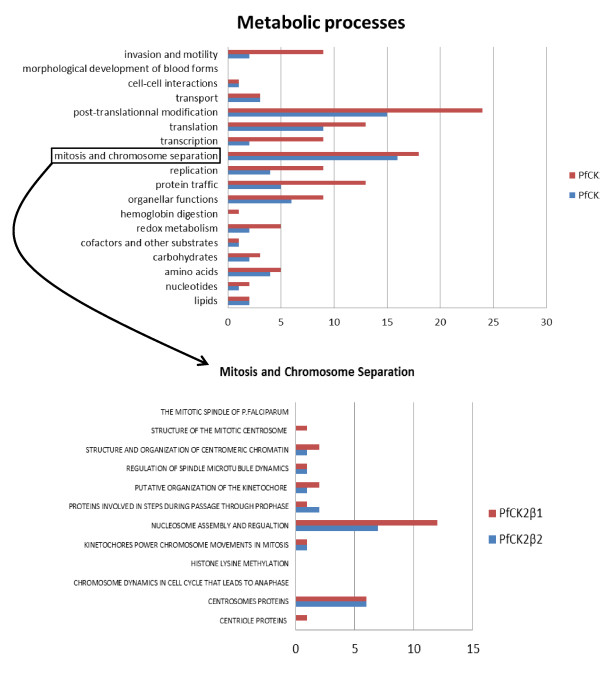
**Potential interactors in various metabolic processes**. **(A) **Histogram representing the number of potential PfCK2β interactors distributed among the metabolic processes described in the Metabolic Pathways of Malaria Parasites website http://sites.huji.ac.il/malaria/. **(B) **Potential interactors in the post-translational modification pathways and the mitosis and chromosome separation pathway. The nucleosome assembly and regulation pathway contained a higher number of proteins co-precipitating with the CK2 regulatory subunits.

We examined the distribution of all other potential interactors among the various parasite metabolic processes described in the Malaria Parasite Metabolic Pathways (MPMP) website (curated by H. Ginsburg; http://sites.huji.ac.il/malaria/) (Figure 4, top panel). Although some pathways were not represented at all in the immunoprecipitates, or had only a very small number of entries, others displayed many hits in both lists.

A different distribution pattern was seen when the same protocol, using the same anti-HA antibody, was applied to parasites expressing other HA-tagged kinases, such as PfCK1 (data not shown); this result lends support to the specificity of the pull-down approach. For both PfCK2β subunits, the highest numbers of potential interactors were found in the processes of 1) post-translational modifications, and 2) mitosis and chromosome separation. As we were aiming to understand the nuclear function of PfCK2, we focused further investigations on the latter process.

Each metabolic process covers several specific pathways. Of the various pathways grouped under the 'mitosis and chromosome separation' process, some contained very few or no hits (Figure 4, bottom panel). By contrast, 12 proteins belonging to the 'nucleosome assembly and regulation pathway' (see Additional file 2, Figure A5) were found in the PfCK2β immunoprecipitates (Table 1). These included both *P. falciparum *nucleosome assembly proteins PfNapL (PFL0185c) and PfNapS (PFI0930c). Co-immunoprecipitation experiments coupled to western-blot analysis established that HA-tagged PCK2α can also pull down both PfNaps (see Additional file 2, Figure A6). Naps are negatively charged proteins that act as histone chaperones and function as histone depositors, allowing ordered formation of chromatin at definite sites. PfNapL and PfNapS proteins interact *in vitro *with *P. falciparum *histones H2A, H2B, H3, and H4, and are *in vitro *substrates for human CK2 [33,34]. A number of histones and their variants, and several proteins thought to be involved in virulence-gene regulation through association with sub-telomeric chromatin at the nuclear periphery, were also identified in the β subunit precipitates. These included two of the four Alba *P. falciparum *proteins that have recently been identified as perinuclear DNA-RNA-binding proteins and thought to play a role in gene regulation in asexual stages [35]; the prototype Alba protein was described as an archeal chromatin protein [36].

**Table 1:** Potential interactors in the nucleosome assembly and regulation pathway.^a^

Protein^b^	PlasmoDB	PfCK2β1HA (3D7/tagged)^c^	PfCK2β2HA (3D7/tagged)^c^
PfCK2α	MAL13P1.237	1/161	1/188

PfCK2β1	PF11_0048	1/31	1/24

PfCK2β2	PF13_0232	0/37	0/33

PfNapL	PFL0185c	0/3	0/3

PfNapS	PFI0930c	0/4	0/2

Histone H2A	PFF0860c	0/35	X^d^

Histone H2A variant	PFC0920w	2/25	2/6

Histone H2B	PF11_0062	2/11	2/3

Histone H2B variant	PF07_0054	0/4	X

Histone H3	PFF0510w	3/13	3/5

Hhistone H4	PF11_0061	2/11	X

High mobility group protein	MAL8P1.72	0/2	X

Chromodomain helicase DNA-binding protein	PF10_0232	0/3	0/6

Smarca-related protein	PFF1185w	0/8	0/2

Structure-specific recognition protein	PF14_0393	0/2	X

PfAlba1	PF08_0074	6/12	6/7

PfrAlba2	MAL13P1.233	0/2	X

PfAlba3	MAL13P1.237	2/9	2/4

PfAlba4	PF10_0063	2/6	X

^a^Proteins found in the HA-PfCK2β1 and HA-PfCK2β2 immunoprecipitates, which are part of the 'nucleosome assembly and regulation pathway' described in the MPMP website, or have been implicated in chromatin dynamics.

^b^Data are taken from Additional Tables A2 and A3, which display the entire list of recovered proteins.

^c^For each column, the unweighted spectrum count is given first for the immunoprecipitate obtained from 3D7 wild-type parasite, and then from the transgenic parasites expressing HA-tagged PfCK2β1or PfCK2β2.

^d^X signifies that the protein was not found in the immunoprecipitate.

### *P. falciparum *Naps, histones and Alba proteins are substrates for PfCK2α *in vitro*

**Figure 5:**
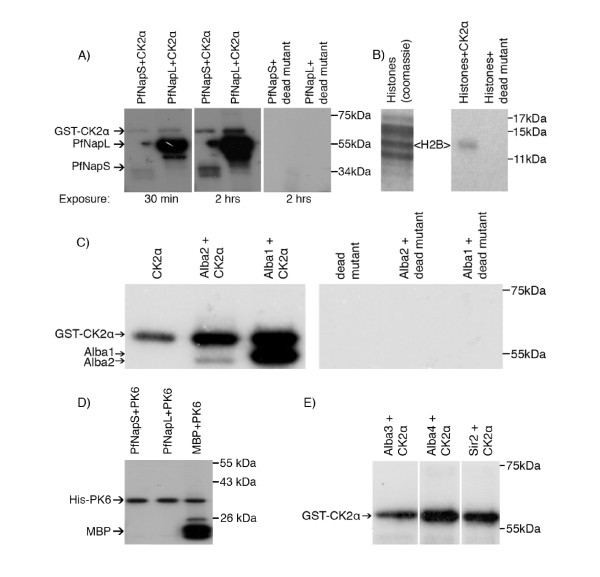
**PfNaps, histones and PfAlba are substrates for recombinant PfCK2α**. *In vitro *kinase assays were performed on purified native parasite histones and recombinant PfNapL, PfNapS, four members of the PfAlba gene family, and Sir2, using active GST-PfCK2α and with a GST-PfCK2α K72M dead mutant. The PfCK2α kinase yielded an autophosphorylation band at 60 kDa, whereas the kinase-dead mutant did not yield any signal with any of the tested substrates. **(A) **GST-PfCK2α phosphorylates PfNapS and PfNapL, with PfNapL giving a much stronger signal. (Left and middle): active GST-PfCK2α; (right): kinase-dead GST-PfCK2α K78M mutant. **(B) **GST-PfCK2α specifically phosphorylated histone H2B (right panel, left lane) in a mixture of native histones purified from the parasite (left panel: Coomassie stain of the purified native histones used for the assay). The PfCK2α kinase-dead mutant did not yield any signal. **(C) **GST-PfCK2α phosphorylated PfAlba1 and PfAlba2 GST fusion proteins, producing signals at 53 and 51 kDa respectively (left panel, bottom bands). (Left panel) Active GST-PfCK2α; (right panel) kinase-dead GST-PfCK2α K78M mutant. **(D) **His-PfPK6 wass unable to phosphorylate PfNapS and PfNapL, but autophosphorylates and phosphorylates MBP. **(E) **GST-PfCK2α is unable to phosphorylate PfAlba3, PfAlba4, and Sir2 GST fusion proteins. Only the GST-PfCK2α autophosphorylation band was visible.

We tested whether recombinant PfCK2α could phosphorylate selected chromatin-associated proteins from the potential PfCK2β interactor list. Kinase assays were performed with glutatione S-transferase (GST)-tagged PfCK2α, using a GST-PfCK2α K72M dead mutant as a negative control (Figure 5). PfCK2α autophosphorylation generated a band of ~60 kDa in size, but the dead mutant did not autophosphorylate, and was unable to phosphorylate exogenous substrates, as previously reported [19]. When subjected to a kinase assay using PfCK2α, both recombinant PfNaps were phosphorylated, with PfNapL (55 kDa) giving a very strong signal (after as short an exposure time as 30 minutes), and PfNapS (37 kDa) appearing as a much less efficient substrate (even after a longer exposure time of 2 hours) (Figure 5A). No phosphorylation of the Nap proteins was seen, either with the PfCK2 kinase-dead-mutant control, nor with the unrelated PK PfPK6 [37,38], despite the ability of the latter enzyme to autophosphorylate and to phosphorylate myelin basic proteins in our assay conditions (Figure 5D).

We next examined the ability of histones to act as substrates for recombinant GST-PfCK2α. Recently, several phosphorylation sites in plasmodial histones have been identified ([39]; Dastidar *et al.*, unpublished data), and because several histones were recovered in the PfCK2β precipitates (Table 1), we thought that PfCK2α might be one of the kinases that can phosphorylate histones. When purified parasite native histones were subjected to a GST-PfCK2α kinase assay, preferential phosphorylation of histone H2B (13 kDa) was seen, whereas the dead-mutant kinase failed to phosphorylate any of the histones (Figure 5B). We also tested in PfCK2α kinase assays all four *P. falciparum *Alba members expressed as GST fusion proteins [35], as well as the nuclear protein PfSir2, which is implicated in establishment of heterochromatin in sub-telomeric region and hence represses *var *gene expression [40-42]. PfAlba3, PfAlba4, and Sir2 were not phosphorylated by GST-PfCK2α (Figure 5E). By contrast, PfAlba1 (55 kDa) and PfAlba2 (52 kDa) gave signals on the autoradiographs after a kinase assay was performed with GST-PfCK2α, whereas none of the substrates subjected to the GST-PfCK2α dead mutant was phosphorylated (Figure 5C).

## Discussion

Our reverse-genetics data strongly suggest that both PfCK2β subunits are essential for parasite viability, indicating that they have non-redundant functions in the parasite. This was unexpected given the dispensability of the β subunits for viability in *S. cerevisiae *(which has two CK2β subunits that can be knocked out individually or simultaneously [14,22,23]) and ib *Schizosaccharomyces pombe *(which had a single β subunit) [43]. However, the single β subunit present in *C. elegans *[21] and in mice [20] is essential. The different interactomes for the CK2β subunits in *S. cerevisiae *[44] are consistent with functional specialization of each β subunit. The immunoprecipitates obtained with PfCK2β1 and PfCK2β2, despite displaying significant overlap in the identity of recovered proteins, also contained hits that were found in only one of the two immunoprecipitates (see Additional file 5, Table A4). This would be consistent with the non-redundancy in function suggested by reverse genetics; however, we do not have sufficient data to draw any firm conclusion in this respect. The dataset presented in the supplementary material (see Additional file 3, Table A2; see Additional file 4, Table A3) is from one of three experiments. Although several hits obtained in the first dataset were confirmed in two further experiments, fewer proteins were recovered in the latter, pre-empting statistical analysis, More work is needed to confirm that individual proteins are truly absent from one of the other PfCK2β interactome. However, the data (see Additional file 3, Table A2; see Additional file 4, Table A3) nevertheless enabled us to propose assignment of specific functions to PfCK2.

Using recombinant proteins in pull-down experiments, we previously found that PfCK2α is able to interact with both β subunits *in vitro *[19]. MS analysis of immunoprecipitated subunits (this study) indicates that these interactions are also found in parasite extracts. CK2 is known to have several functions in various organisms [11,12], and the glimpse into the interactomes of the *P. falciparum *CK2 regulatory subunits provided by our study is consistent with their involvement in a number of cellular processes. Implementing the same protocol with different HA-tagged kinases such as PfCK1 (data not shown) yielded protein repertoires that were clearly distinct from those described here for the PfCK2β subunits, lending support to the specificity of the approach. Potential PfCK2 interactors were found in 17 out of 18 metabolic processes described on the MPMP website. Many processes displayed only a very small number of hits; whether or not these represent genuine interactors will require in-depth investigations of each individual case.

By contrast, other processes and pathways exhibited a large proportion of their proteins recovered in the immunoprecipitates, suggesting that a number of distinct functional complexes had been pulled down. A striking example is the 'nucleosome assembly and regulation' pathway, many components of which were found in both PfCK2β1 and PfCK2β2 immunoprecipitates (Table 1). This included both PfNAPs, many histones, and several proteins involved in chromatin remodelling (ISWI, CHD1, high mobility group protein, and structure-specific recognition protein). Interestingly, all recovered proteins were members of the so-called 'activating factors' in this pathway, whereas none of the 'inactivating factors' were present in the immunoprecipitates (see Additional file 2, Figure A5), which again is consistent with the specificity of the approach. Some of these interactions were validated by coupled co-immunoprecipitation/western-blot analysis (see Additional file 2, Figure A6) and *in vitro *kinase assays (Figure 5).

Both PfNap proteins were phosphorylated by recombinant PfCK2α. In yeast, phosphorylation of Nap1by one of the two catalytic subunits of CK2 is implicated in cell cycle progression, as cells expressing CK2 phosphorylation site Nap1 mutants display a lengthened S-phase and a shortened G1 phase, together resulting in a delay in mitosis. Interestingly, these mutants retained the wild-type ability to interact with histones [45]. It has been proposed that phosphorylation of the PfNaps by human recombinant CK2 may affect their affinity for histones [34]. PfCK2 might also act on PfNaps *in vivo*, thus affecting their function, and in accordance with this hypothesis, recent global phosphoproteomic analyses of parasite extracts [46,47] found both PfNaps be phosphorylated on acidic peptides predicted to be CK2 substrates by the NetPhos tool (Table 2).

**Table 2:** Phosphopeptides identified in parasite extracts in two published phosphoproteome studies [46,47].^a^

Gene name	PlasmoDB ID	Phosphopeptide^b^	NetPhos2 CK2 score^c^	Ref
PfNapL	PFL0185c	EISSLLEsIKIDDDK		[46]
**PfNapL**	**PFL0185c**	**EISsLLESIK**	**0.842**	[47]
PfNapL	PFL0185c	IDDDKMtDLTEEQK		[46,47]
PfNapL	PFL0185c	VPNsNVIK		[46,47]
PfNapS	PFI0930c	DRsENsVENTDPK		[46,47]
**PfNapS**	**PFI0930c**	**sDLDNEIPK**	**0.851**	[47]
PfNapS	PFI0930c	HVtFDNNQEK		[47]
PfNapS	PFI0930c	NPIAAVTHNRsDLDNEIPK		[46]
PfAlba1	PF08_0074	MtNYVNYGAK		[47]
PfAlba1	PF08_0074	CGsTVItDQYVsGQDNsEHVVQEK		[47]
**PfAlba1**	**PF08_0074**	**GSTVITDQYVsGQDNsEHVVQEK**	**0.602**	[46]
**PfAlba1**	**PF08_0074**	**EMtPEEIVNSR**	**0.727**	[46,47]
**PfAlba2**	**MAL13P1.233**	**NEDKKsGDEEEEEEEEEEDEENNK**	**0.991**	[46]
PfAlba2	MAL13P1.233	PGsTKsDTKLENEIR		[46]
**PfAlba2**	**MAL13P1.233**	**sDtKLENEIR**	**0.772**	[47]
PfH2B	PF11_0062	YDsYGLYIFK		[47]
PfH2B	PF11_0062	sMNIMNsFLVDTFEK		[47]

^a^Phosphosites predicted to be CK2 substrates by analysis using the KinasePhos2 tool http://kinasephos2.mbc.nctu.edu.tw/ are highlighted in ^b^The phosphosite in the peptides are indicated by lowercase s or t. bold characters, and the score is indicated in the fourth column.

^c^Only the sites experimentally shown to be phosphorylated are included here; note that there are additional predicted CK2 sites in these proteins.

We found that PfCK2α preferentially phosphorylated histone H2B *in vitro *in a mixture of native histones extracted from the parasite. Histone post-translational modifications such as methylation and acetylation play key roles in the control of expression of virulence genes (for example, the *var *family) in malaria parasites [48]) Similarly, histone phosphorylation is known to play an important regulatory role in other eukaryotes [49], and in yeast, CK2 phosphorylates histone H4 at serine 1 as part of a DNA damage-signaling pathway [50]. Although previous studies have identified few phosphorylation marks in *P. falciparum *histones, we recently characterized several novel phosphorylation marks on the parasite's histones (Dastidar *et al.*, unpublished). Whether PfCK2 is responsible for phosphorylation of any of these sites *in vivo*, and the functional consequences of these phosphorylation events, remain to be investigated. Nevertheless, taken together, our data strongly suggest that PfCK2-dependent phosphorylation may play a role in chromatin dynamics.

We also tested other known nuclear proteins with a potential role in gene regulation to see if they could be substrates for the kinase. Of all the proteins tested, recombinant PfAlba1 and PfAlba2 were found to be substrates for the PfCK2α *in vitro *(Figure 5), and PfAlba1 was found to be phosphorylated in parasite extracts on several residues [46,47], including one in an acidic stretch consistent with CK2 substrate preferences (Table 2). By contrast, recombinant PfAlba3, PfAlba4, and PfSir2 were not phosphorylated by the kinase (Figure 5E). PfAlba1 and PfAlba2 belong to the same phylogenetic cluster, which is distinct from that containing PfAlba3 and PfAlba4 ([51,52]), consistent with their different ease if phosphorylation by PfCK2. Moreover, the former two PfAlbas possess an arginine- and glycine-rich RGG domain in addition to the Alba domain. It has been shown in *S. cerevisiae *that phosphorylation of the RGG domains affects protein localization and transport between the nucleus and cytoplasm [53]. Given that PfAlba1 and PfAlba2 concentrate at the perinuclear space in ring-stage parasites, but expand to the cytoplasm of trophozoites and schizonts [51], it is possible that phosphorylation by PfCK2 may contribute to the regulation of their nucleocytoplasmic transport.

Several proteins involved in mitosis and chromosome separation and in DNA replication and DNA damage pathways (for example, several replication factor C subunits, ATP-dependent DNA helicase, DNA ligase 1) were also pulled down. Implication of CK2 in the DNA damage response is well documented in other systems [12], and a DNA repair pathway has been characterized in *P. falciparum *[54], of which the DNA polymerase and DNA ligase components can be retrieved in the CK2β immunoprecipitates (see Additional file 3, Table A2; see Additional file 4, Table A3; see Additional file 5, Table A4). Because DNA repair is markedly reduced in the highly drug-resistant W2 isolate, which displays a higher rate of drug-resistance acquisition than do drug-sensitive strains [55], it might be of interest to compare CK2 activity in the W2 versus drug-sensitive clones.

Although many of the proteins identified in the immunoprecipitates were of nuclear origin, a number of potential interactors were associated with other cellular compartments. Some of the recovered proteins were putative or established Maurer's cleft proteins (for example, Maurer's cleft 2 transmembrane protein; Pfmc-2TM), proteins involved in invasion and motility processes (for example, glutamate-riche protein, merozoite surface protein 2 precursor, RhopH3, and other rhoptry-associated proteins) or in cell-cell interaction processes (for example, mature parasite-infected erythrocyte surface antigen, PfEMP2 and rifins). Likewise, several proteins and enzymes involved in redox metabolism were recovered (thioredoxin, thioredoxin-related protein, thioredoxin peroxidase, glutathione peroxidase, glutathione S-transferase, ribonucleotide reductase). This diversity is consistent with our IFA, western blot, and immuno-EM data localizing all the three subunits to both the nuclear and cytoplasmic compartments. Although these results require confirmation by other approaches, and despite the fact that we cannot exclude false positives in our interactome list, they are consistent with an involvement of PfCK2 kinase in varied cellular functions in the parasite.

The availability of parasite lines expressing individual tagged PKs for the almost entire *P. falciparum *kinome [46] now allows implementation of a kinome-wide investigation of kinase function based on the interactomics approach used here for PfCK2. Preliminary data obtained with other PKs suggest that distinct functions can therefore be assigned to different enzymes on the basis of specifically pulled-down protein repertoires.

## Conclusion

In conclusion, we have identified the PfCK2 complex as a kinase distributed to different cellular compartments. Our data point to PfCK2 as a pleiotropic kinase with various cellular functions. In this study, we explored potential nuclear functions of the kinase, and we propose a role for PfCK2 in modifying a number of chromatin-related proteins, several of which are also substrates for the catalytic subunit of PfCK2 *in vitro*. Furthermore, reverse genetics data show that all three subunits of the kinase are essential for the viability of the blood-stage parasite, confirming its potential as target for chemotherapeutic intervention. This study paves the way for a kinome-wide interactomics-based approach to elucidate PK function in malaria parasites.

## Methods

### Construction of the knockout plasmids pCAM-BSD-KOPfCK2β1 and pCAM-BSD-KOPfCK2β2

Fragments from central region of the PfCK2β1 and PfCK2β2 ORFs were amplified by PCR and inserted between the *Bam*HI and *Not*I sites of the pCAM-BSD plasmid [31], which contains the *Aspergillus terreus *blasticidin-*S*-deaminase gene, whose gene product confers resistance to blasticidin (see Additional file 1, table A1, for cloning primers).

### Construction of the complementation plasmids pCHD-PfCK2β1 and pCHD-PfCK2β2

A plasmid for *in vivo *episomal expression of PfCK2β1 and PfCK2β2 subunits were constructed as follows. The full-length coding sequences were first inserted between the *Bgl*II and *Not*I sites of plasmid pHGB [56], and then transferred into plasmid pCHD-1/2 [56] by a clonase reaction (Gateway system, Invitrogen) according to the manufacturer's instructions. Plasmid pCHD-1/2 includes a cassette encoding human dihydrofolate reductase, conferring resistance to the antifolate drug WR99210. Parasites that were transfected with both a KO plasmid and a complementation plasmid were cultured under double-drug selection (see below).

### 3'-tagging of plasmids

The 3' end of the PfCK2β2 coding sequence (573 bp, omitting the stop codon) was amplified by PCR using primers incorporating *Pst*I and *Bam*HI restriction sites and inserted between the same sites of the pCAM-BSD-HA plasmid [57]. For *pfCK2β1*, the 3' end of the gene (540 bp) was amplified, then the PCR product was treated with the restriction enzymes *Pst*I and *Bgl*II and inserted between the *Pst*I and *Bam*HI sites of pCAM-BSD-HA.

### Parasite culture and transfection

Four *P. falciparum *strains (3D7A [58] and three HA-tagged strains of PfCK2 subunits: PfCK2αHA, PfCK2β1HA and PfCK2β2HA) [19] were grown in human erythrocytes as described previously [59]. The medium for the HA-tagged lines was supplemented with blasticidin (2.5 μg/ml; VWR International Ltd., West Chester, PA, USA). Cultures of the *P. falciparum *clone 3D7A were maintained at 37°C in RPMI 1640 medium (Gibco) supplemented with 25 mmol/l sodium bicarbonate, 2 mmol/l L-glutamine, 300 mmol/l hypoxanthine, 10 μg/ml gentamicin, and lipid-rich bovine serum albumin (BSA) (AlbuMAX II; Sigma-Aldrich, St Louis, MO, USA). Cultures were seeded at 5% hematocrit volume and maintained at a parasitemia of 1 to 10% with daily changes of medium. The incubator was flushed with a gas mixture containing 5% CO_2_. For transfection, asexual blood-stage parasites were synchronized by sorbitol treatment [60] so as to result in the majority being ring-stage parasites. Forty-eight hours later, the ring-stage parasites were transfected by electroporation with 100 μg of purified plasmid DNA in buffer (Cytomix; Gibco-BRL, Grand Island, New York, NY, USA) as described previously [61]. Blasticidin (2.5 μg/ml) was added to the culture medium to select for transformed parasites. Parasites under double selection had 5 nmol/l WR99210 added to the culture medium in addition to blasticidin. Parasites were maintained in this supplemented medium from 2 days post-transfection.

### DNA extraction and Southern blotting

Parasite cultures were lysed in 0.15% saponin. The parasite pellets were resuspended in cold phosphate-buffered saline, and treated with 150 μg/ml proteinase K and 2% SDS at 55°C for 2 to 3 hours. Total DNA was extracted in phenol:chloroform:isoamyl alcohol (25:24:1) and precipitated in ethanol and 0.3 mol/l sodium acetate. DNA was digested with *Cla*I and *Eco*RI for PfCK2β1, and with *Cla*I and *Nco*I for PfCK2β2.

### Western-blot analysis

Cytoplasmic and nuclear extracts were prepared as described previously [41,62]. Using standard procedures. SDS-PAGE was performed and DNA was transferred to nitrocellulose membranes, which were probed using either commercially available antibodies against HA-tag (ab9110; 1:1000 dilution), histone H3 (ab1791; 1:2500 dilution), *P. falciparum *aldolase (ab38905; 1:1000) (all Abcam, Cambridge, MA, USA) or subunit-specific antibodies generated in rabbits against the PfCK2β1-derived peptide DSNKDLQDSKSDKS (1:500 dilution) or the PfCK2β2-derived peptide DEINRDSEEMYKNK (1:1000 dilution (both BioGenes GmBH, Berlin, Germany) After probing with appropriate secondary antibodies conjugated to horseradish peroxidase (GE Healthcare Life Sciences, Princeton, NJ, USA), the membranes were developed with a commercial substrate (Super Signal West Pico Chemiluminescent Substrate; Thermo Fisher Scientific Inc., Rockford, IL, USA) in accordance with the manufacturer's recommendations.

### Immunofluorescence assays

IFAs were performed on synchronized stages (ring, trophozoite, and schizont) of the three HA-tagged strains of the PfCK2 subunits as described previously [56]. The fixed cells were incubated with primary antibodies anti-HA (ab9110; 1:2000 dilution) and anti-Exp2 [32] (1:800 dilution) (both Abcam). After incubating with appropriate secondary antibodies, the cells were examined under a microscope (Nikon Instruments, Tokyo, Japan).

### Immuno-gold electron microscopy

Infected erythrocytes were fixed in 1% glutaraldehyde in RPMI-HEPES buffer for 1 hour at 4°C. After washing, polymerization in agar (Type IX; Sigma-Aldrich), and dehydration with ethanol, the samples were transferred into embedding resin (LR-White; London Resin Company Ltd, Reading, Berkshire, UK), and polymerized for 12 hours at 4°C, and cut. Ultrathin sections of the sample were collected and mounted on Cu/Pd grids. Free aldehyde groups were blocked by incubating the samples for 5 minutes in 50 mmol/l NH_4_Cl. Sections were blocked in phosphate-buffered saline containing 5% (v/v) normal goat serum, 1% (w/v) BSA and 0.01% (v/v) Tween-80. The washed grids were incubated for 2 hours in antiserum diluted in phosphate-buffered saline containing 1% (v/v) normal goat serum, 1% (w/v) BSA and 0.01% (v/v) Tween-80 and anti-HA antibody (ab9110; Abcam). Samples were washed and incubated for 45 minutes with 12-nm gold-labeled goat anti-rabbit IgG (111-205-144; Jackson Immunoresearch Laboratories, Inc., West Grove, PA, USA). Washed sections were stained for 15 minutes with aqueous 4% uranyl acetate followed by staining for 2 minutes with 1% lead citrate. Sections were analyzed using an electron microscope (JEM-1200EX; JEOL Ltd, Tokyo, Japan) at 120 kV.

### Parasite proteins extraction and immunoprecipitation

Parasite cultures were lysed in 0.15% saponin and pellets were stored at -80°C. Proteins were extracted on ice using mammalian protein extraction reagent (M-PER) (Profound™ Mammalian HA-tag IP/Co-IP application; Thermo Fisher Scientific) with protease inhibitors (1:25; Complete Protease Inhibitor Cocktail; Roche Diagnostics, Basel, Switzerland) and benzonase nuclease (1:500; Novagen, Darmstadt, Germany). The lysates were cleared by centrifugation at 11,180 *g *for 15 minutes at 4°C, and the concentration of proteins measured by the Bradford assay. Proteins were loaded on a spin column with a pre-inserted frit, preventing bead loss between washes, and 6 μl (50% v/v_ of anti-HA crosslinked onto sepharose beads were added (both included in the kit used for protein extraction; Thermo Fisher Scientific). The columns were then transferred to a rocker platform and incubated overnight. The flow-through was collected by centrifugation at 300 *g *for 10 seconds at room temperature. The columns were washed three times using the Tris-buffered saline (TBS) provided in the kit, followed by a short burst of centrifugation (300 *g *for 10 seconds). Finally, any proteins bound to the beads were eluted using boiled 4× Laemmli buffer, and recovered by centrifugation (15,600 *g *for 3 minutes). Aliquots of protein extracts and flow-through liquid were taken before and after immunoprecipitation to monitor the yield.

### Mass spectrometry analysis

Samples were separated by SDS-PAGE on 10% polyacrylamide gel, which was then stained with Coomassie blue (Biosafe; Bio-Rad Laboratories, Inc., Hercules, CA, USA), and later placed in 10% ethanol and 1% acetic acid. Each lane was removed from the gel and cut into eight slices, then the proteins were digested in the gel using trypsin As follows. Briefly, the samples were reduced in 10 mmol/l dithioerythritol (DTE) and alkylated in 55 mmol/l iodoacetamide (IAA), the gel pieces were dried. The samples were incubated with 12.5 ng/ml trypsin overnight at 37°C. The tryptic peptides were extracted from the gel slices, dried, and resuspended in 2% acetonitrile (ACN):0.1% formic acid (FA) for liquid chromatography/tandem mass spectrometry (LC-MS-MS) analysis

A mass spectrometer (LTQ Orbitrap XL (Thermo Fischer Scientific) equipped with an ultraperformance LC (UPLC) system (NanoAcquity; Waters Ltd, Elstree, Hertfordhshire, UK) was used. Peptides were trapped in a custom-made precolumn (Magic C18 AQ stationary phase, 5 μm diameter, 200Å pore, 0.1 × 20 mm, Michrom Bioresource) and separated in a custom-made main column (Magic C18 AQ, 3 μm diameter, 100Å pore, 0.75 × 150 mm), using a run of 53 minutes and a gradient of H2O:ACN:FA 98%:2%:0.1% (solvent A) and ACN:H2O:FA 98%:1.9%:0.1% (solvent B). The gradient was run at a flow rate of 250 nl/min as follows: 100% A for 3 min, 30% B within 36 min, 47% B within 14 min, 90% B within 5 min held for 5 min and 100% A for 17 min. The MS/MS was operated in an information-dependent mode, in which each full MS analysis was followed by 10 MS/MS acquisitions, during which the most abundant peptide were selected for collision-induced dissociation (CID) to generate tandem mass spectra. The normalized collision energies were set to 35% for CID.

Data search was performed using Mascot software (version 2.3; Matrix Science Inc., Boston, MA, USA) and Proteome Discoverer (v.1.1; Thermo Fisher Scientific), and the sequences searched against a concatenated database consisting of non-redundant *Plasmodium *database (PlasmoDB, version 6.4; http://plasmodb.org) and the reversed-sequence version of the same database and common contaminant proteins. Finally, results were imported into Scaffold (version 3_00_8 Proteome Software; http://proteomesoftware.com/) for validation of protein identification, normalization, and comparison of spectral count. Peptide identifications were accepted if they could be established at a probability of greater than 95% as determined by the ProteinProphet algorithm [63]. Protein identifications were accepted if they were assigned at least two unique, validated peptides, and could be established with at least 99% probability as determined by the ProteinProphet algorithm [64].

### Kinase assays

The GST-PfCK2α and GST-PfCK2α-dead K72M mutant fusion proteins [19] were purified from *E. coli *(BL21 Gold strain) as described previously [65]. PfCK2α kinase activity was determined by measuring ^33^P or ^32^P incorporation using γ[^33^P]-ATP or γ[^32^P]-ATP. All the substrates (Alba1, Alba2, Alba3, Alba4, PfSir2; kindly provided by C. Scheidig-Benatar), and extracted *P. falciparum *histones were treated with active PfCK2α and dead mutant as described previously [65]. Samples were run on a 16% gel, which was dried and exposed to film for autoradiography.

## Competing interests

The authors declare that they have no competing interests.

## Authors' contributions

EGD, GD, and ZH equally contributed to design of the study and acquisition of data. EGD participated in generating immunofluorescence and western-blot data about the localization of the kinase subunits, and in drafting of the manuscript along with ASc and CD. GD generated and analyzed all the data from the interactome study along with RH and MM. ZH generated all transgenic lines and PfCK2 expression plasmids used in the study. DDS and EGD performed all the kinase assays. ACl generated the immuno-electron microscopy data and analyzed them along with EGD. ACh and ASh provided the, respectively, the recombinant Alba proteins and Naps protein used in the study. JJLR participated in design of experiments and provided critical comments about the manuscript. ASc and CD conceived the study, participated in its design and coordination. All authors read and approved the final manuscript.

## Supplementary Material

Additional file 1 **Additional Table A1**. Table of primers used in this study. Click here for file

Additional file 2 **Additional Figures A1-A6**. Additional Figures A1-A6 and their legends. Click here for file

Additional file 3 **Additional Table A2**. List of proteins identified by mass spectrometry analysis of protein complexes immunoprecipitated from the parasite line expressing hemagglutinin-tagged PfCK2β1. Click here for file

Additional file 4 **Additional Table A3**. List of proteins identified by mass spectrometry analysis of protein complexes immunoprecipitated from the parasite line expressing hemagglutinin-tagged PfCK2β2. Click here for file

Additional file 5 **Additional Table A4**. Mass spectrometry data from immunoprecipitates obtained with PfCK2β1 and PfCK2β2 hemagglutinin-tagged transgenic lines, highlighting the significant overlap in the identity of recovered proteins, and the occurrence of potential interactors that are unique to either PfCK2 regulatory subunit. Note that the data are not sufficient to draw statistically supported conclusions about the possible restriction of proteins to the interactome list of one or the other β subunit, and that this table must be considered as a preliminary dataset only. Click here for file
