# Supplementary material for: Involvement of Plasmodium falciparum protein kinase CK2 in the chromatin assembly pathway
Source: BMC Biol. 2012 Jan 31;10:5. doi: 10.1186/1741-7007-10-5 (PMC3296614; doi:10.1186/1741-7007-10-5)
Supplement: Additional file 1 — Additional Table A1. Table of primers used in this study. [file 1741-7007-10-5-S1.DOC]

| **Primer name** | **Primer Sequence** | **Primer Direction** |
| --- | --- | --- |
| **CK2β1ForEco** | GGGGGAATTCATGGAAAATAGTGATTCGAATAAAGAC | Forward |
| **CK2β1RevSal** | GGGGGTCGACTTACGTTTCAGAAATTTGTAGTTCTTCC | Reverse |
| **CK2β2ForBam** | GGGGGGATCCATGGAGTTTGTTTCAAACGATGAAAG | Forward |
| **CK2β2RevSal** | GGGGGTCGACTCATTGACACTCTTCAGAGGATTCCG | Reverse |
| **CK2β2RSpe** | GGGGACTAGTTCATTGACACTCTTCAGAGGATTCCG | Reverse |
| **CK2β1FBgl** | GGGGAGATCTATGGAAAATAGTGATTCGAATAAAGAC | Forward |
| **CK2β2shortForBam** | GGGGGGATCCATGGAAGCAACAGTGTCTTGGATTG | Forward |
| **CK2β13primeRev** | GCATTAAAATATGAGATGTACAC | Reverse |
| **CK2β23primeRev** | CAAACTATGTCAACTGTTTTGGG | Reverse |
| **CK2a5primeFor** | GAGACAGGAATAATG | Forward |
| **CK2β15primeF** | CTTAAGTGTTAATCGG | Forward |
| **CK2β25primeF** | GGCATAGGAATATTTAAC | Forward |
| **CK2β1KOForBgl** | GGGGAGATCTTAAGAAAATAGTGATTCGAATAAAGAC | Forward |
| **CK2β1KORevNot** | GGGGGCGGCCGCATATACATGAACTTTGGC | Reverse |
| **CK2β2KOForBam** | GGGGGGATCCTAAGATGAAAGTGCAGATGACATAATC | Forward |
| **CK2β2KORevNot** | GGGGGCGGCCGCATATAATAATGGGCTTAAAAATTTAG | Reverse |
| **CK2β1TAGForPst** | GGGGCTGCAGGCTGGGGATGCACCTGAAG | Forward |
| **CK2β1TAGRevBglII** | GGGGAGATCTCGTTTCAGAAATTTGTAG | Reverse |
| **CK2β2TAGForPst** | GGGGCTGCAGGTTCCTCATTTTAAGAAGC | Forward |
| **CK2β2TAGRevBam** | GGGGGGATCCTTGACACTCTTCAGAGGATTCC | Reverse |
| **pCAMBSDFor** | TATTCCTAATCATGTAAATCTTAAA | Forward |
| **pCAMBSDRev** | CAATTAACCCTCACTAAAG | Reverse |
| **T7** | GCTAGTTATTGCTCAGCGG |  |
| **SP6** | ATTTAGGTGACACTATAG |  |

**Table A1: Oligonucleotide primers used in plasmid construction and PCR diagnostics.** Restriction enzyme recognition sequences are underlined.
