# Supplementary material for: Involvement of Plasmodium falciparum protein kinase CK2 in the chromatin assembly pathway
Source: BMC Biol. 2012 Jan 31;10:5. doi: 10.1186/1741-7007-10-5 (PMC3296614; doi:10.1186/1741-7007-10-5)
Supplement: Additional file 2 — Additional Figures A1-A6. Additional Figures A1-A6 and their legends. [file 1741-7007-10-5-S2.PDF]

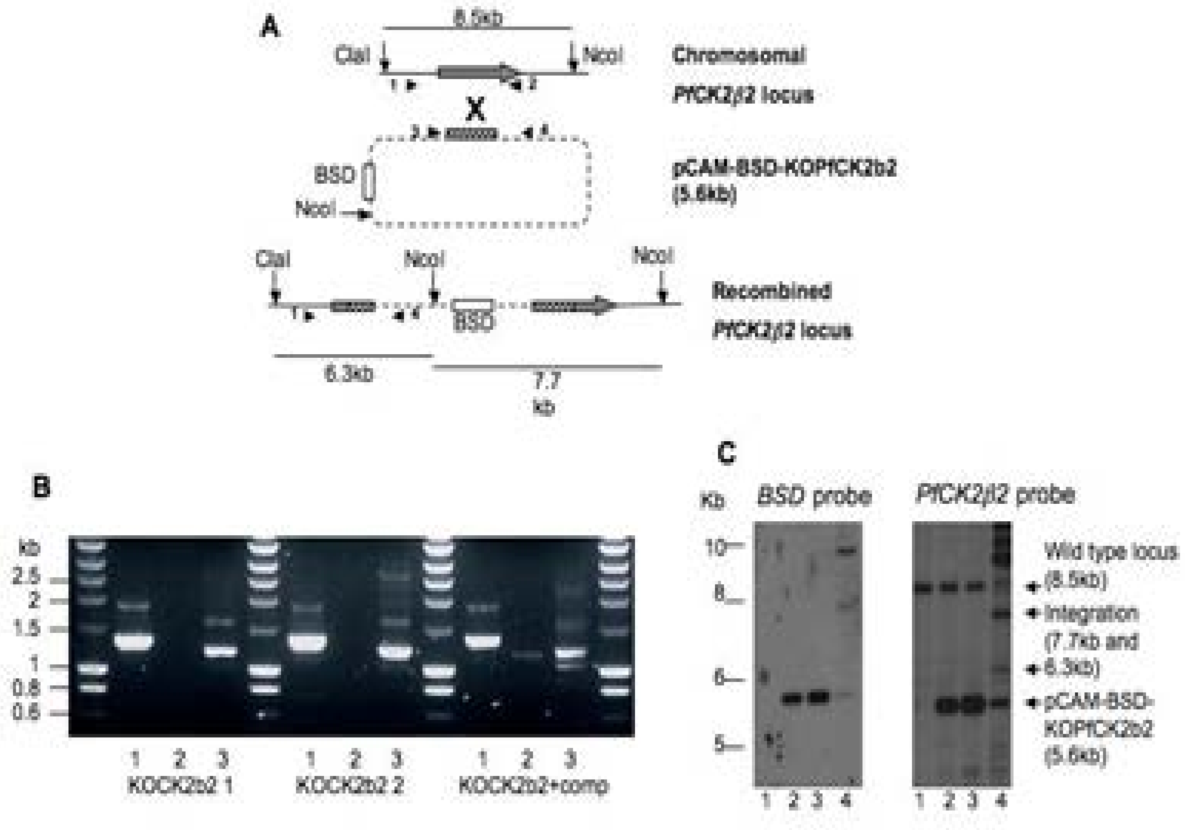

	Primers	Expected size of amplified band (kb)
**Wild type (lane 1)**	**1 + 2**	**1.3**
**5’ integration (lane 2)**	**1 + 4**	**1.1**
**Plasmid (lane 3)**	**3 + 4**	**1.1**

Additional Fig. A1

For the legend, please see next page

Legend to Additional Figure A1. Knockout attempt of *PfCK2β2*

3D7 parasites transfected with pCAM-BSD-KOPfCK2β2 alone or alongside pCHD-PfCK2β2 were analysed by PCR and Southern blotting.

Panel A: Diagram showing the locations of the primers used for PCR screening, and the recognition sites for the restriction enzymes used to cut the gDNA to give a diagnostic pattern of bands for analysis by Southern blotting.

Panel B: PCR screening of gDNA from untransfected 3D7 parasites, two separate pCAM-BSD-KOPfCK2β2-transfected cultures (KOCK2β2 1 and KOCK2β2 2), and parasites transfected with both the knockout plasmid and the complementation plasmid (KOCK2β2 + comp).
1: amplification of the wild-type *pfck2*β2 locus.
2: amplification over the 5' integration boundary.
3: amplification of the insert in the pCAM-BSD-KOPfCK2β2 plasmid.

Panel C: Evidence of integration is seen only in doubly-transfected parasite line (KOCK2β2 + comp). Parasite gDNA was also analysed by Southern blotting. The restriction enzymes ClaI and NcoI were used to digest the gDNA, and the fragments were analysed by Southern blotting using *BSD* and *PfCK2β2* as probes.

1: untransfected 3D7
2: KOCK2β2 1
3: KOCK2β2 2
4: KOCK2β2 + comp.

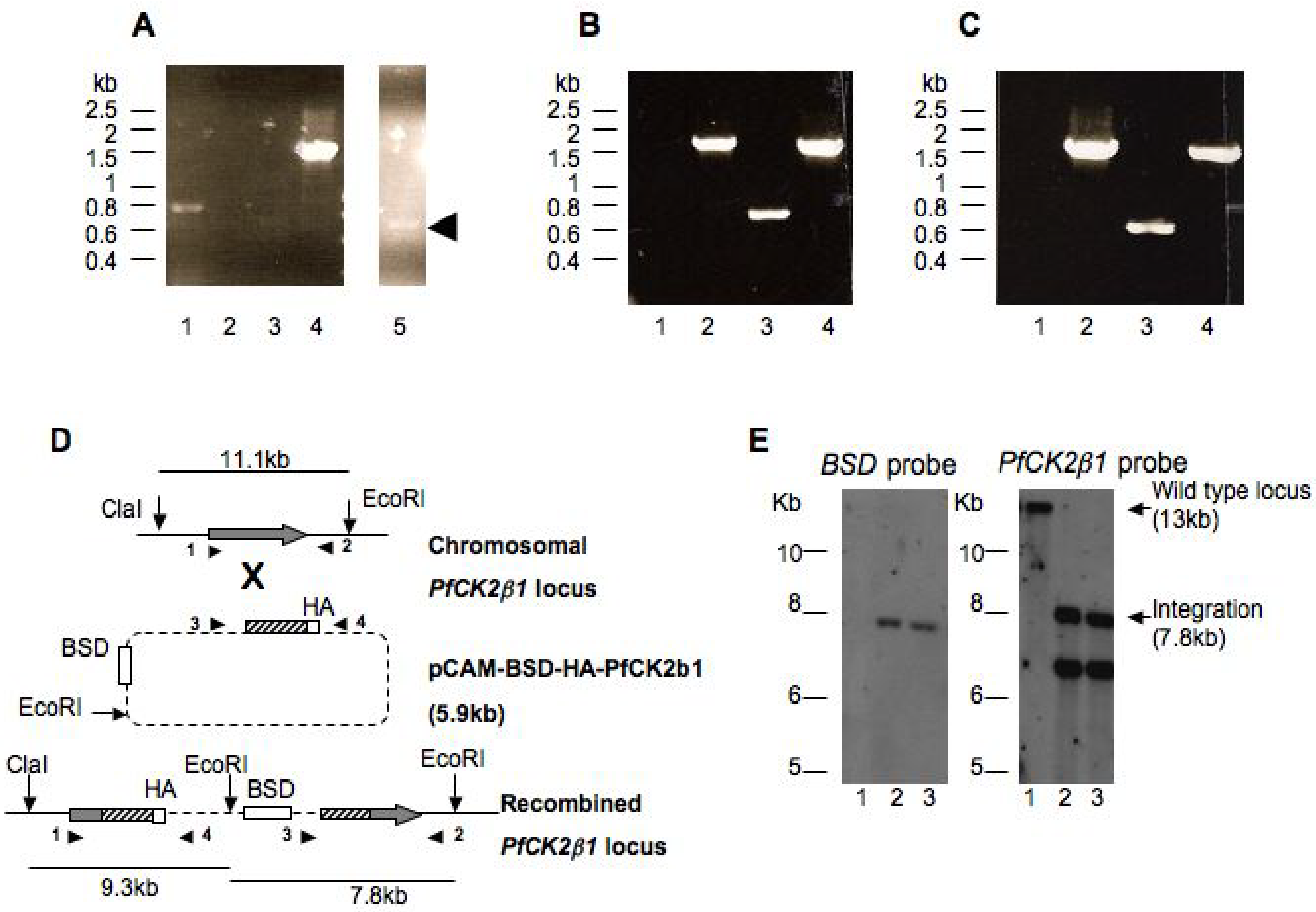

Additional Fig. A2

For the legend, please see next page

Legend to Additional Figure A2. C-terminal tagging of PfCK2β1.

A: PCR screening for integration in DNA from parasites transfected with pCAM-BSD-HA-PfCK2β1 revealed the presence of parasites in which integration events had occurred (lane 3: 722bp. This lane is shown overexposed, where the 3' integration band, which is indicated with an arrowhead, can more clearly be seen). Lane 1: amplification of the wild-type locus, lane 2: attempted amplification of the 5' integration locus, lane 3: 3' integration locus, land 4: diagnostic band for the presence of the plasmid.

B, C: PCR screening for integration in gDNA of parasite lines derived by limiting dilution (B: clone C9, C: clone E8) revealed that the wild-type band had been lost (lane 1: 804bp) and only the integration bands (lane 2: 1673bp, lane 3: 722bp) were seen. These clonal lines were further analysed by Southern blotting (Panel E).

D. Schematic of the chromosomal gene locus, the pCAM-BSD-HA-PfCK2β1 plasmid and the recombined locus, and shows the locations of oligonucleotide primers used for the PCR screens (Panels A-C). Oligonucleotides are listed in Additional Table A1. The location of the restriction enzyme recognition sites is depicted and the expected sizes of the fragments of DNA after restriction digestion are shown.

E. Digested DNA was analysed by Southern blotting, using PfCK2β1 and BSD sequences as probes. DNA from wild-type 3D7 parasites (lane 1), and PfCK2b1HA clonal lines C9 (lane 2) and E8 (lane 3), was digested using the restriction enzymes EcoRI and ClaI. The wild-type locus, recognised by the PfCK2b1 probe, disappears in the clonal lines, indicating that integration has occurred.

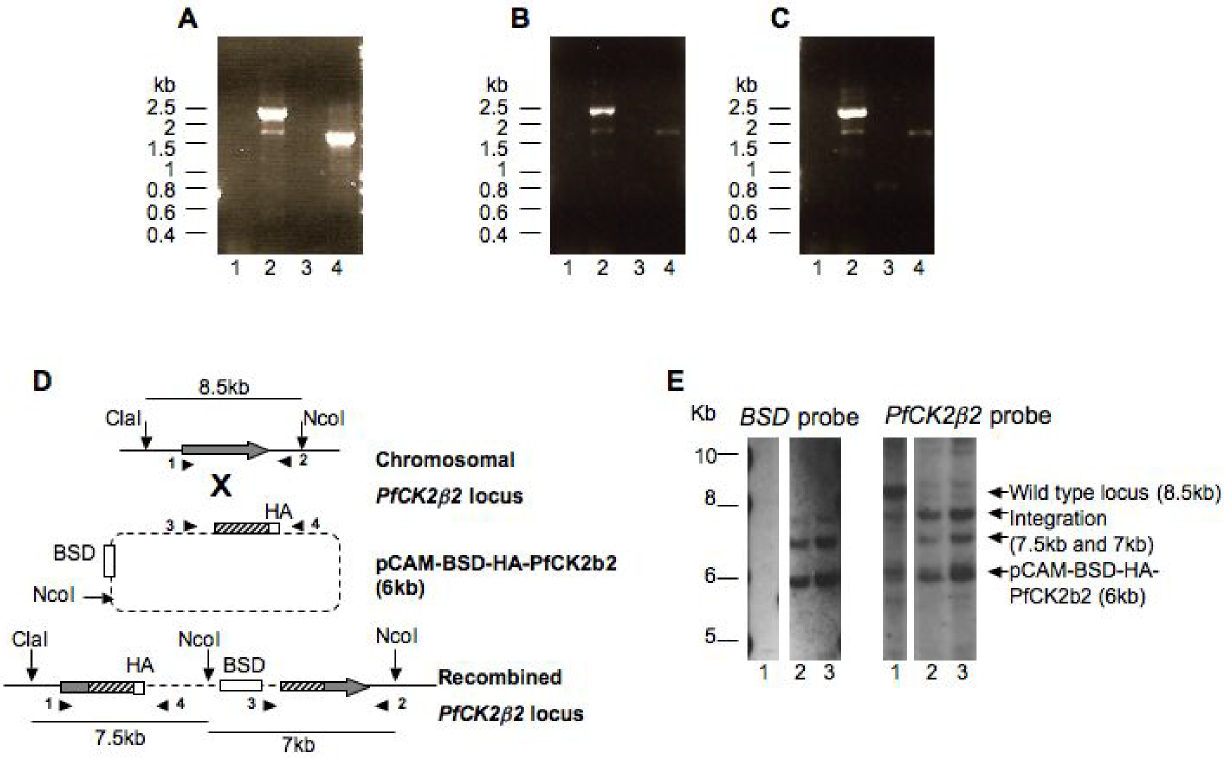

Additional Fig. A3

For the legend, please see next page

Legend to Additional Figure A3. C-terminal tagging of PfCK2β2

A. PCR screening for integration in gDNA from parasites transfected with pCAM-BSD-HA-PfCK2β2 revealed the presence of parasites in which integration events had occurred (lane 2: 2255bp).

B and C. PCR screening for integration in gDNA of clonal cultures derived by limiting dilution (B: clone D5, C: clone E2) revealed that the wild type band had been lost (lane 1: 1483bp) and the integration bands were seen (lane 2: 2255bp, lane 3: 822bp). These two clonal lines were further analysed by Southern blotting (Panel E).

D. Schematic of the chromosomal gene locus, the pCAM-BSD-HA-PfCK2b2 plasmid and the recombined locus, and shows the locations of oligonucleotide primers used for the PCR screens (Panels A-C). Oligonucleotide identities are listed in Table S1. The location of the recognition sites for the restriction enzymes NcoI and ClaI are depicted, and the expected sizes of the fragments of gDNA after restriction digestion are shown.

E. Fragments of total DNA resulting from restriction endonuclease digestion were analysed by Southern blotting, using *BSD* and *PfCK2β2* as probes. Total DNA from wild type 3D7 parasites (lane 1), and PfCK2b2HA clonal lines D5 and E2 (lanes 2 and 3), was digested using the restriction enzymes NcoI and ClaI. The wild type locus, recognised by the *PfCK2β2* probe, disappears in the clonal lines, and integration bands of the expected sizes are seen, indicating that the plasmid

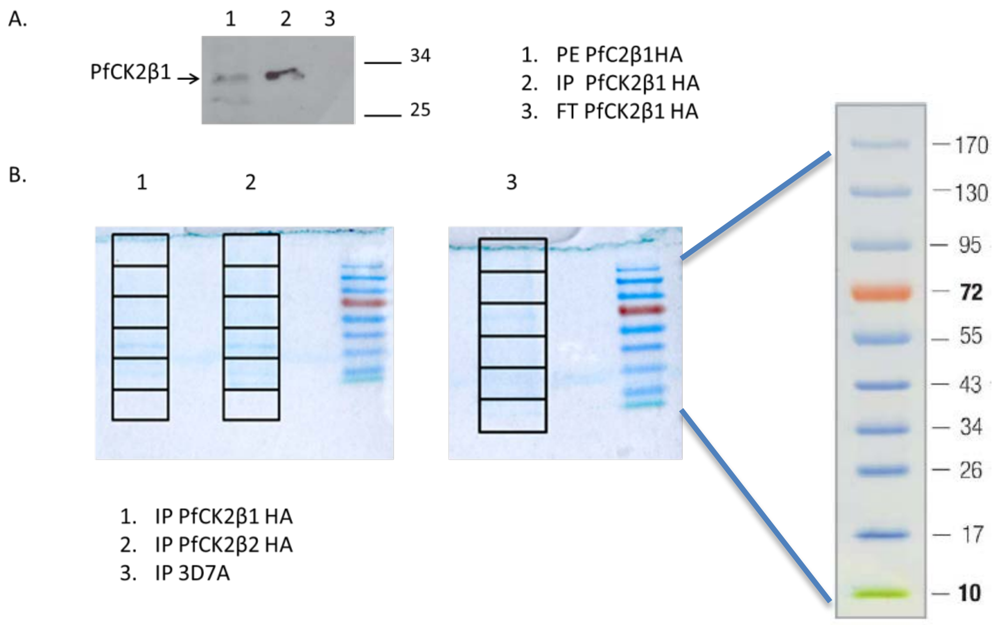

Additional Fig. A4

Legend to Additional Figure A4: Control of IP efficiency and gel slices analysed by mass spectrometry.

A. PfCK2β1 Western-blot. Protein extract (PE) before immunoprecipitation (IP) with anti HA (lane 1), protein extract immunoprecipitated with anti HA (lane 2) and flow through (FT, lane 3) samples were separated by SDS-PAGE, transferred to nitrocellulose and probed with an antibody directed against PfCK2β1.

B. Biosafe Coomassie Blue-stained polyacrylamide-SDS gel run after the IP. Gel slices excised for subsequent MS analysis are indicated by black boxes, and molecular masses of the marker are indicated to the right.

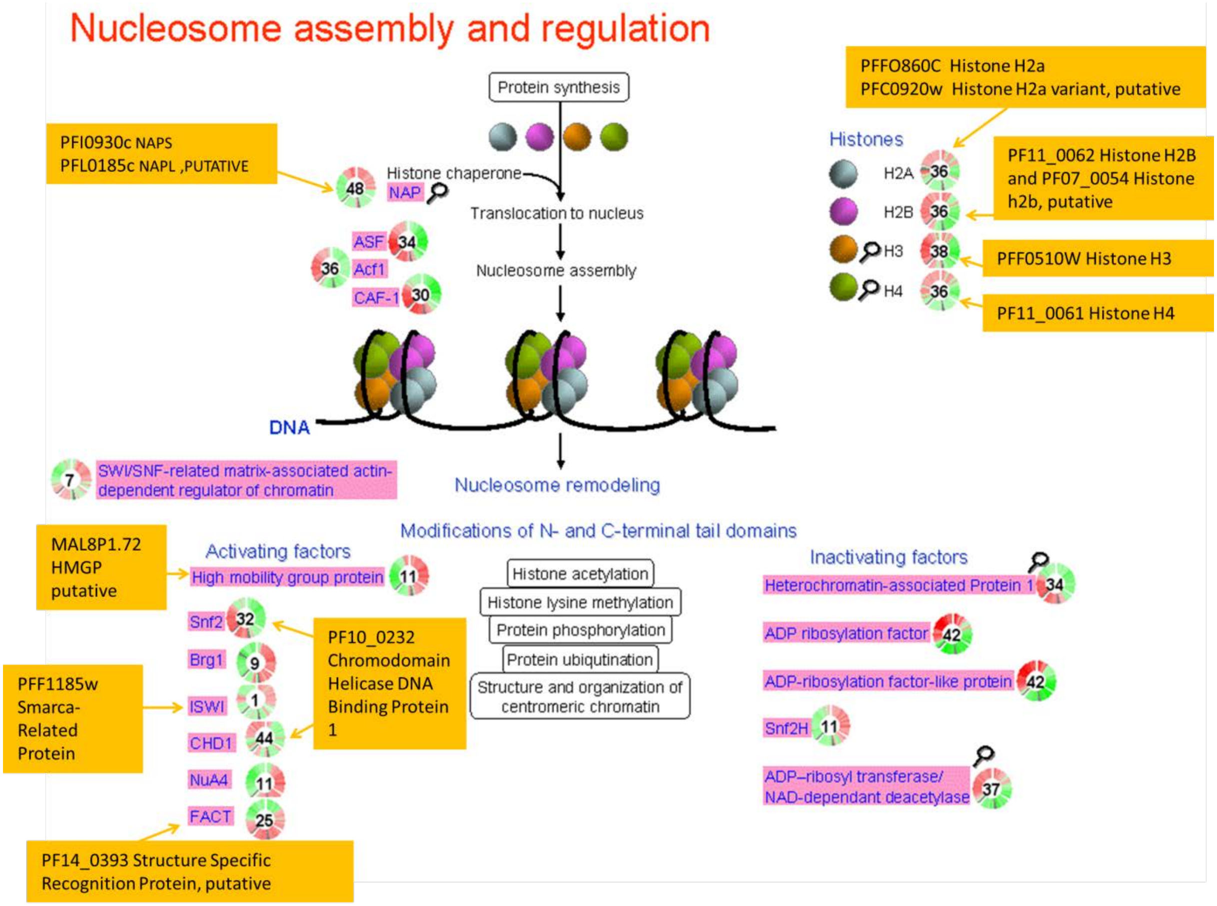

Additional Fig. A5

Legend to Additional Figure A5. The nucleosome assembly pathway

Adapted from the MPMP website (http://sites.huji.ac.il/malaria/maps/NucleoAssembly.html).
Proteins recovered in the PfCK2β immunoprecipitates are indicated in orange boxes.

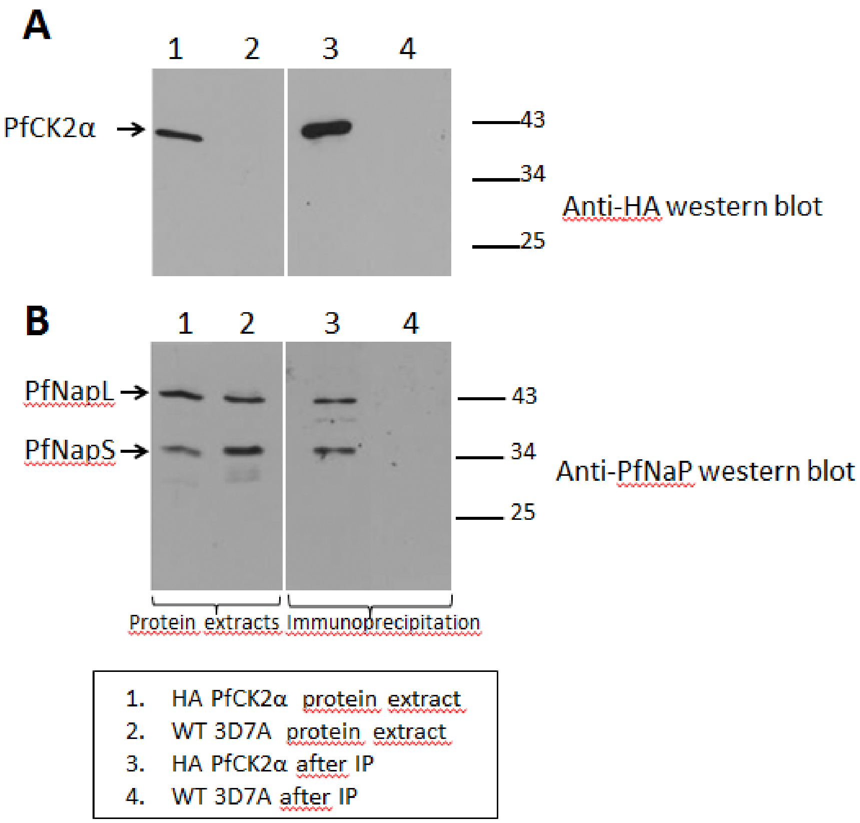

Additional Fig. A6

Legend to Additional Figure A6. Interaction between recombinant PfCK2a and PfNaps.

Proteins from asynchronous cultures of parasites expressing HA-tagged PfCK2α (lanes 1 and 3) or 3D7A (lanes 2 and 4) were immunoprecipitated using an anti-HA antibody cross-linked to agarose bead. Initial protein extract from PfCK2α-HA (lane 1) and 3D7 wild-type (lane 2), as well immunoprecipitates from PfCK2α-HA (lane 3) and 3D7 (lane 4), were fractioned by SDS-PAGE, transferred and probed.

A. Membrane probed with anti-HA revealed the presence of PfCK2α in the parasite extract (lane 1) and immunoprecipitate (lane 3).

B. Membrane probed with a mixture of anti-PfNapL and anti-PfNapS antibodies.
